# Community-acquired pneumonia in diabetic patients is characterised by a distinct pathogen spectrum and enhanced inflammation: results from CAPNETZ, a prospective observational cohort study

**DOI:** 10.1007/s15010-025-02659-w

**Published:** 2025-10-12

**Authors:** Belén Millet Pascual-Leone, Facundo Fiocca Vernengo, David Hillus, Charlotte Wernicke, Gopinath Krishnamoorthy, Jan Rupp, Gernot Rohde, Mathias W. Pletz, Martin Witzenrath, CAPNETZ-study group, A. Fuchs, G. Paul, M. Ayoub, A. Prasse, W. Bauer, E. C. Diehl-Wiesenecker, N. Galtung, C. Kodde, Y.-M. Stoppe, C. Boesecke, S. Breitschwerdt, D. Benke, S. Schmager, A. Grünewaldt, J. Wheeler, B. Schaaf, J. Kremling, M. Kolditz, B. Schulte-Hubbert, J. Ronczka, A. Seeger, J. Kohlhäufl, D. Stolz, S. Fähndrich, M. Panning, M. Unnewehr, R. Lim, M. Hoeper, I. Pink, N. Drick, T. Fühner, T. Steinberg, G. Barten-Neiner, W. Kröner, O. Unruh, N. Adaskina, F. Eberhardt, T. Illig, N. Klopp, B. T. Schleenvoigt, A. Moeser, D. Drömann, P. Parschke, K. Franzen, F. Waldeck, B. Gebel, N. Käding, S. Boutin, J. Schneider, J. Erber, F. Voit, D. Heigener, I. Hering, W. Albrich, F. Rassouli, B. Wirth, C. Neurohr, A. Essig, S. Stenger, M. Wallner, H. Burgmann, L. Traby, L. Schubert, Norbert Suttorp, Leif Erik Sander, Andreas Vestergaard Jensen, Bastian Opitz, Charlotte Thibeault

**Affiliations:** 1 Department of Infectious Diseases, Respiratory Medicine and Critical Care, Charité – Universitätsmedizin Berlin, corporate member of Freie Universität Berlin and Humboldt-Universität zu Berlin, Augustenburger Platz 1, 13353 Berlin, Germany; 2 Department of Endocrinology and Metabolism, Charité – Universitätsmedizin Berlin, corporate member of Freie Universität Berlin and Humboldt-Universität zu Berlin, Berlin, Germany; 3 CAPNETZ Stiftung, Hannover, Germany; 4 Infectious Diseases Clinic and Institute of Medical Microbiology, University Hospital Schleswig-Holstein, Luebeck, Germany; 5 German Center for Lung Research (DZL), Berlin, Germany; 6 Department of Medicine, Pulmonary, Critical Care and Sleep Medicine, Philipps University of Marburg (UMR), Member of the German Center for Lung Research (DZL), Marburg, Germany; 7 Biomedical Research in Endstage and Obstructive Lung Disease Hannover (BREATH), Member of the German Center for Lung Research (DZL), Hannover, Germany; 8 Institute for Infectious Diseases and Infection Control and Center for Sepsis Care and Control, Jena University Hospital, Friedrich-Schiller-University, Jena, Germany; 9 Department of Pulmonary and Infectious Diseases, Nordsjællands Hospital, Hillerød, Denmark; 10 Institute of Health at Charité-Universitätsmedizin Berlin, Berlin, Germany

**Keywords:** Diabetes mellitus, Community-acquired pneumonia, Pathogen spectrum, Enterobacteriaceae

## Abstract

**Purpose:**

Diabetes mellitus (DM) is a relevant risk factor for enhanced susceptibility to and adverse outcomes in infections, including community-acquired pneumonia (CAP). We aimed to characterise clinical outcomes, inflammatory and organ failure markers and microbial etiologies in diabetic (DM+) versus non-diabetic (DM−) patients in a European CAP cohort.

**Methods:**

Comparative analyses using data from the CAPNETZ multicenter, prospective, observational study including 13,611 patients with CAP enrolled between 2002–2022, with and without a history of DM, were conducted.

**Results:**

Seventeen percent (2310/13,611) had a history of DM (DM+). Compared to DM− patients, DM+ patients had a higher 180 days mortality rate following CAP (13% (292/2310) vs. 7% (766/11,301), *p* < 0.0001) and higher C-reactive protein and leucocyte counts (median CRP 97 mg/L (IQR: 31–202) vs. 86 mg/L (IQR: 24–190), *p* < 0.0001; median leucocyte count 12/nl (IQR: 9–16)vs. 11/nl (IQR: 8–15), *p* < 0.0001). Pathogens were identified in 23.4% (540/2310) of the DM+ and 21.7% (2414/11,301) of the DM− patients (*p* = 0.03), respectively. Overall, pathogen distribution differed between the two groups, with higher frequencies of Enterobacteriaceae in the DM+ group (13.0% (70/539) vs. 8.0% (194/2414), *p*_adj_ < 0.01).

**Conclusions:**

CAP in DM+ is characterised by a distinct microbial spectrum and enhanced inflammation. While further studies are needed to elucidate the clinical impact of our findings, we recommend early and comprehensive CAP pathogen testing in DM+ patients.

**Supplementary Information:**

The online version contains supplementary material available at 10.1007/s15010-025-02659-w.

## Introduction

Lower respiratory tract infections (LRIs), including community-acquired pneumonia (CAP), are the world’s most deadly transmittable diseases, ranked as the fourth leading cause of death by the WHO in 2019 [1] and associated with a global mortality rate of 27.7 per 100,000 across all ages in 2021 [2]. Effective antibiotic therapy is crucial to mitigate adverse outcomes of CAP. The pathogen distribution in CAP forms the rational basis for local empirical antibiotic therapy guidelines. As microbiological testing is typically not performed in the outpatient setting and pathogen detection rates are only between 20 and 40% in those tested, most CAP patients are treated empirically [3, 4]. Therefore, continuous investigation into likely pathogens across various patient populations remains essential to optimise treatment recommendations. It is widely accepted that the causative pathogen spectra in CAP vary in accordance to the geographical region, observation period and pathogen identification methods used [5]. CAP aetiology also depends on host characteristics. Cardiac, cerebrovascular, chronic respiratory and kidney disease, nursing home residence, prior antimicrobial therapy and immunosuppression have been associated with a higher risk of pneumonia with multidrug-resistant pathogens and/or Enterobacteriaceae and non-fermenting gram-negative bacilli such as *Pseudomonas aeruginosa* [6, 7]. Both groups include potentially multidrug-resistant (MDR) pathogens, which may limit the efficacy of empirical antimicrobial therapy.

Diabetes mellitus (DM) is among the most frequent comorbidities in CAP populations [8, 9]. Previous analyses identified a prevalence of known DM (irrespective of diabetes type) of 15% in the full German Community-Acquired Pneumonia Competence Network (CAPNETZ) cohort [10]. Evidence suggests that diabetic (further called DM+) patients are more susceptible to certain types of infections (e.g., LRIs, urinary tract, and skin infections) compared to non-diabetic (DM−) individuals [11, 12]. A systematic review found DM to be associated with increased post-discharge and hyperglycaemia with increased in-hospital mortality following CAP [13]. While it is plausible that the presence of DM may differentially influence susceptibility to specific microbes, including opportunistic pathogens [11, 12], there is, to the best of our knowledge, no large study examining CAP aetiologies in DM+ versus DM− patients in Europe [14, 15].

The aim of this work was to compare the pathogen spectrum in DM+ versus DM− patients with CAP along with clinical characteristics, outcomes, parameters of inflammation and organ dysfunction in DM+ vs. DM− patients in the CAPNETZ cohort.

## Methods

### Capnetz cohort

Data was obtained from patients enrolled into the CAPNETZ study [16], a multicenter prospective cohort study on CAP conducted in hospitals and private practices in Germany, Switzerland, Austria, the Netherlands, Denmark and Italy [17]. The study was conducted in accordance with the Declaration of Helsinki, as well as guidelines of Good Clinical Practice. It was approved by the institutional ethics board of the Hannover Medical School, Germany (Ethics approval No. 301-2008). Inclusion criteria were: age ≥ 18 years, informed consent, evidence of lung infiltrate by imaging, and at least one of the following: active coughing, purulent sputum, positive auscultation findings, or fever. Exclusion criteria were: hospitalisation for more than 48 h before CAP diagnosis and newly diagnosed, active pulmonary tuberculosis. Demographic and clinical data, including previous medication, therapies, and comorbidities, laboratory data, results from microbiological and virological testing, and data on predefined outcomes (ICU admission within 28 days, death from any cause within 180 days from enrolment into the study) were collected in a case report file.

**Fig. 1:**
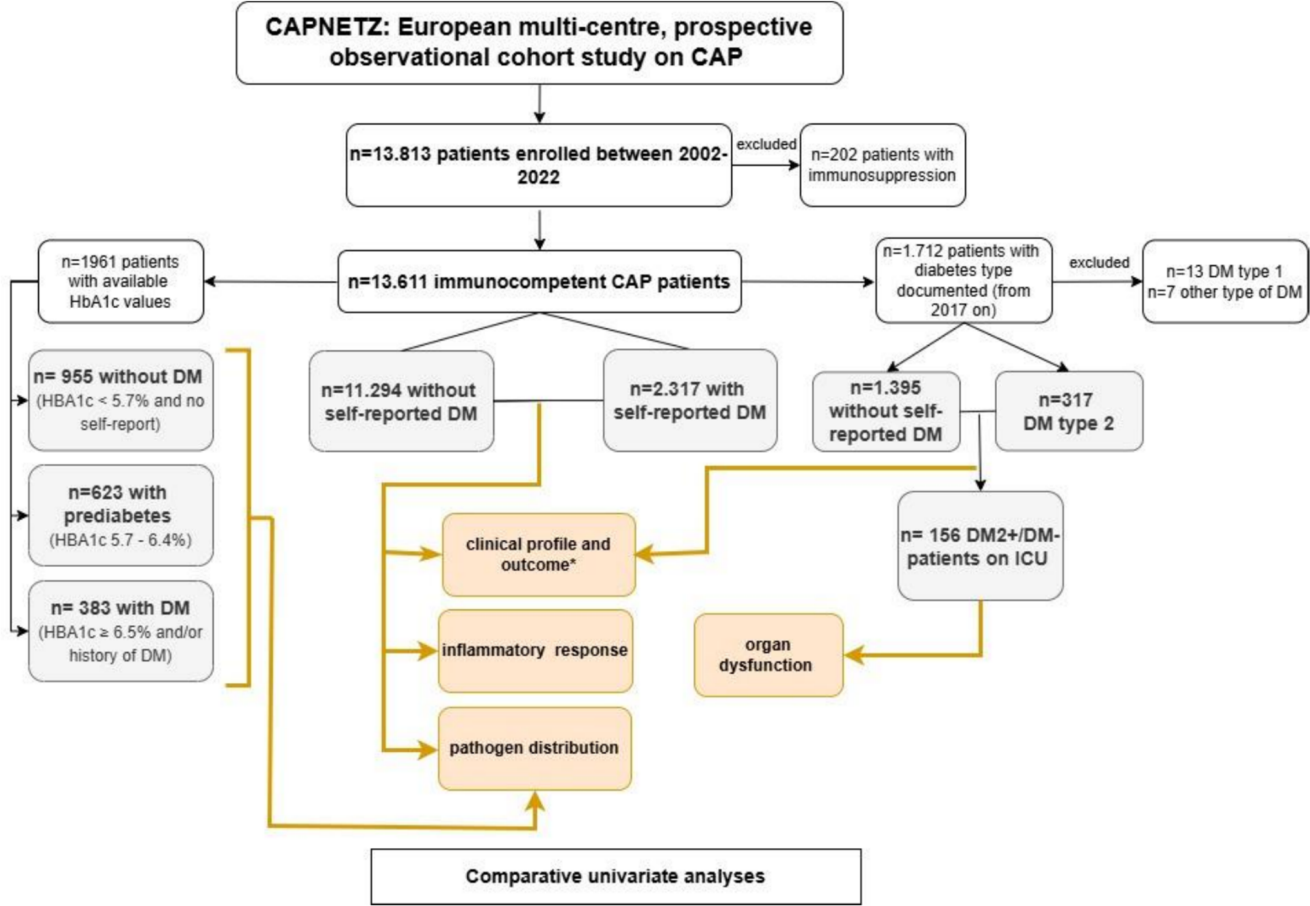
Study flow chart. All patients included into the CAPNETZ study between 2002–2022 were considered for this analysis. After exclusion of immunosuppressed patients, 13,611 patients with (DM+) and without (DM−) self-reported diabetes were included retrospectively into the main cohort for comparative analyses of clinical profile and outcome, inflammatory response and pathogen spectrum. HbA1c values were available for *n* = 1961 patients. In this cohort, pathogen spectrum was analysed comparatively in DM−, DM+ and prediabetic patients. The type of diabetes was documented as part of the protocol from 2017 on. In *n* = 156 patients with type 2 diabetes or DM− admitted to the intensive care unit (ICU subcohort), markers of organ dysfunction were comparatively assessed. Yellow colour indicates analyses performed: univariate analyses were performed and adjusted for multiple testing, where applicable. * regarding outcome, logistic regression analyses were performed

Patients enroled between October 1st, 2002 and June 30th, 2022 were included into this retrospective analysis (see Fig. 1). Patients with immunosuppression (any of: HIV infection, cytostatic therapy within the last 28 days, neutropenia, steroid therapy (>/= 20 mg prednisolone-equivalent/day), or immunosuppressive therapy after organ or bone-marrow transplantation within the preceding three months) were excluded. The CRB-65 score was used to assess CAP severity [18].

### Standard laboratory parameters

Laboratory parameters were obtained as part of routine clinical diagnostics within the first 48 h following patient enrolment. We selected available parameters that best reflect (1) the host immune response and (2) organ dysfunction and sepsis severity, inspired by components of the Sequential Organ Failure Assessment (SOFA) score including PaO₂/FiO₂ ratio (Horovitz index), bilirubin, creatinine, thrombocyte count, lactate, and confusion at admission as a proxy for central nervous system dysfunction.

### Pathogen identification

Methods used for microbiological diagnosis and laboratory processing procedures have been described previously [6, 19, 20]. In brief, all respiratory specimens and blood cultures collected at the time of inclusion were immediately processed in the local microbiological laboratories of the participating clinical centers. From 2017 onwards, multiplex PCR analysis was performed additionally to detect the following pathogens from sputum/nasopharyngeal swabs: adenovirus, Human bocavirus, Human coronavirus 229E/HKU1/NL63/OC4, enterovirus, influenza virus A/A H1N1 pdm09, influenza virus B, Human metapneumovirus, parainfluenza virus 1/2/3/4, parechovirus, rhinovirus, *Mycoplasma pneumoniae,* and respiratory syncytial virus A/B. In addition, specific PCRs for *Chlamydophila pneumoniae*, *Legionella pneumophila*, and *Bordetella pertussis* were performed in all patients. Detection of SARS-CoV-2 infection was carried out using rapid antigen testing and/or an updated multiplex PCR method (Siemens Healthineers, Eschborn, Germany) introduced in 2020. The microbiological diagnosis in the CAPNETZ cohort was established for patients who tested positive by PCR and/or culture diagnostics from respiratory samples and/or positive blood cultures in patients with moderate to severe disease (both performed in local laboratories associated with the respective hospitals), and/or urinary antigen testing for *L. pneumophila* and *S. pneumoniae*. Pathogens were considered the causative organisms for CAP according to the criteria published by Krüger et al. [20].

The identified pathogens were categorised into the following seven biologically relevant groups: (1) *S. pneumoniae*, (2) *Haemophilus influenzae*, (3) Enterobacteriaceae (including *Citrobacter spp*., *E. coli*, *Enterobacter spp*., *Hafnia alvei*, *Klebsiella oxytoca*, *K. pneumoniae*, *Klebsiella* spp., *Morganella morganii*, *Proteus mirabilis*, *P. vulgaris*, *Serratia marcescens, Serratia* spp.), (4) non-fermenting gram-negative bacteria (including *Acinetobacter spp., Pseudomonas aeruginosa, Pseudomonas spp., Stenotrophomonas maltophilia*), (5) atypical bacteria (including *L. pneumophila*, *M. pneumoniae* and *C. pneumoniae*), (6) *Staphylococcus aureus* and (7) viruses (including *influenza A and B, SARS-CoV-2, Human coronavirus HKU1, Human coronavirus OC43, parainfluenza virus 2, 3 and 4, adenovirus, enterovirus, respiratory syncytial virus A and B, rhinovirus, Human metapneumovirus A and B, Human bocavirus*). Due to low sample sizes, we summarised all other microbes identified under (8) “other pathogens”.

### Diabetes mellitus and subgroups definitions

History of DM and, for patients enroled from 2017 onwards, type of DM, was documented according to self-report. See Fig. 1 for definition of subgroups. A subgroup analysis was conducted among patients with documented DM type, comparing individuals with type 2 diabetes (DM2) to DM−, while excluding all other types of DM due to low sample sizes. This group is referred to as the “diabetes type 2 subcohort” (DM2 subcohort). Because parameters related to organ dysfunction and sepsis were only consistently documented in critically ill patients, the analysis of these variables was restricted to individuals from the DM2 subcohort admitted to the intensive care unit (ICU)—the “ICU type 2 diabetes subcohort” (ICU DM2).

Additionally, glycated haemoglobin (HbA1c) values were available for a subgroup of 1961 patients from a previous CAPNETZ project and were included as a separate subcohort—the “HbA1c subcohort”. HbA1c was measured as previously described [10]. DM was defined according to the 2025 American Diabetes Association diagnostic criteria [21] as HBA1c ≥ 6.5% (≥48 mmol/mol) as measured upon inclusion and/or self-reported diagnosis of DM. Prediabetes was defined as HBA1c 5.7–6.4% (39–47 mmol/mol) and no DM was defined as HBA1c < 5.7% (<39 mmol/mol) with no self-reported DM diagnosis [21]. To account for the inaccuracy of self-reporting, we performed a sensitivity analysis of pathogen distribution between DM−, prediabetic and DM+ patients in the HbA1c subcohort.

### Statistical analysis

Data are shown as percentages, relative frequencies, medians and interquartile ranges (IQRs) or averages with standard deviation (SD), depending on the underlying distribution. No imputation of missing data was performed. Group comparisons were assessed using the Wilcoxon or the Mann–Whitney U test for continuous, or the Chi-square test for categorical data and a P-value of less than 0.05 was considered significant. The Pearson correlation coefficient was used to analyze the relation between continuous variables. P-values were adjusted for multiple testing when comparing every pathogen group against all other pathogen groups with the Bonferroni-Sidak method. To account for the interdependence of clinical variables, we performed a logistic regression on the outcomes ICU admission versus not within 28 days and death from any cause versus not within 180 days within the whole cohort using the following predictors: age, sex, BMI, DM, malignant disease, chronic cardiac disease, chronic cerebrovascular disease, chronic kidney disease and chronic respiratory disease. Clinical data were analysed using Jmp Pro, version 18.2 (SAS Institute Inc, USA) and GraphPad Version 10 (GraphPad Prism, USA).

## Results

### Clinical characteristics

A total of 13,611 patients were included in this analysis. The study flow chart is shown in Fig. 1. Of all patients, 17% (2310/13,611) had self-reported DM, irrespective of type. Clinical characteristics of all patients, and comparisons between DM+ (all types) and DM− are shown in Table 1. DM2 was the most prevalent of all DM types in those with available information (94%, 317/337), DM type 1 accounted for 3.8% (13/337). We observed similar differences regarding clinical characteristics between DM2 + and DM− patients as between DM+ and DM− in the main cohort. (Supplementary Table 1).

**Table 1:** Clinical characteristics of all, DM+ and DM− patients in the CAPNETZ-cohort between 2002–2022

	All patients (*n* = 13,611)	Diabetes (*n* = 2310)	No diabetes (*n* = 11,301)	*p*-value
Age in years (Mean (SD))	61 (18)	71 (12)	59 (18)	<0.0001
Male Sex at birth (% (*n*/*N*))	57 (7772/13611)	63 (1444/2310)	56 (6328/11301)	<0.0001
BMI (kg/m^2^), Median (IQR), available *n*	25 (22–29), 13,221	28 (24–32), 2226	25 (22–28), 10,995	<0.0001
Nursing home residency (%, *n*/available *N*)	6 (805/13599)	11 (244/2307)	5 (561/11292)	<0.0001
Cigarette smoking during the last 12 months (%, *n*/available *N*)	29 (3821/13316)	20 (454/2222)	30 (3367/11094)	<0.0001
Chronic heart failure (%, *n*/available *N*)	17 (2244/13572)	34 (791/2308)	13 (1453/11264)	<0.0001
Chronic respiratory/pulmonary disease (%, *n*/available *N*)	36 (4912/13611)	43 (986/2310)	35 (3926/11301)	<0.0001
Vascular/Cerebrovascular disease (%, *n*/available *N*)	16 (2159/13572)	29 (678/2301)	13 (1481/11271)	<0.0001
Chronic kidney disease (%, *n*/available *N*)	9 (1272/13566)	22 (517/2303)	7 (755/11263)	<0.0001
Previous antibiotic therapy within the last 4 weeks (%, *n*/available *N*)	24 (3257/13544)	16 (373/2287)	26 (2884/11257)	<0.0001
Malignant disease (%, *n*/available *N*)	10 (1366/13611)	12 (268/2310)	10 (1098/11301)	<0.05
Oxygen Therapy (%, *n*/available *N*)	5 (642/13611)	7 (169/2310)	4 (473/11301)	<0.0001

Available *n* is indicated where variables were not available for all patients. *P*-values comparing diabetic and non-diabetic patients (based on self-reporting) were calculated using the Wilcoxon test for medians, and the unadjusted Chi-square test for frequencies of comorbidities. All clinical variables shown were significantly different between diabetic and non-diabetic patients

*DM+* patients with a history of diabetes mellitus, *DM−* patients without a history of diabetes mellitus, *IQR* interquartile range, *SD* standard deviation, *BMI* body mass index

### Outcome analysis

**Fig. 2:**
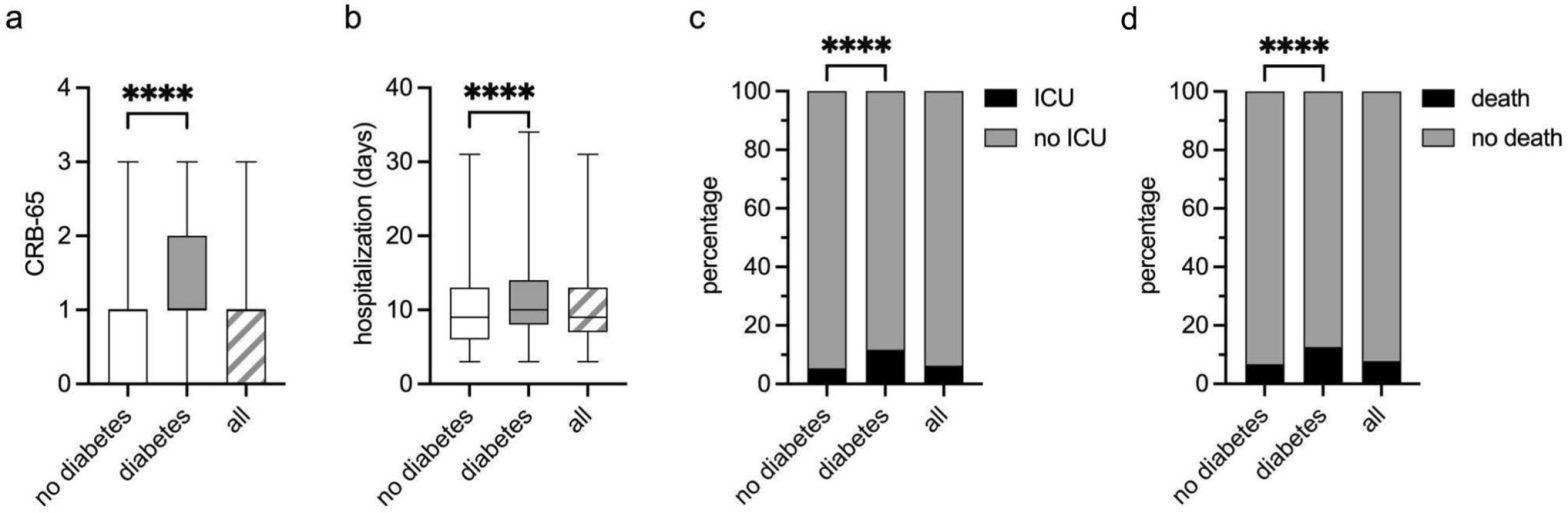
Outcome DM− versus DM+ patients. **a, b** Box plots showing the distribution (median and IQR) of CRB-65 scores (**a**) and hospitalization days (**b**) in patients without (DM−) and with a history of diabetes mellitus (DM+) and all patients (available 12,425/13,611 for (**a**) and 10,259/10,312 for (**b**)). Whiskers represent the 2.5–97.5 percentiles. **c, d** Percentages of ICU admissions within 28 days (**c**) and death within 180 days (**d**), both from any cause following CAP in DM− vs DM+ patients, as well as all patients in the CAPNETZ cohort (available *n* = 9361/13,611 for (**c**) and 13,611/13,611 for (**d**)). Mann–Whitney statistical analysis was used for (**a**) and (**b**), and the Chi-square test was used to analyze the shown groups in (**c**) and (**d**). ****: *p* < 0.0001. CRB-65: pneumonia severity index, ICU: intensive care unit (see methods)

The distribution of CRB-65 scores differed significantly between the two groups (DM+: median: 1, IQR: 1–2 vs. DM−: 1, IQR: 0–1) (Fig. 2a). DM+ patients were hospitalised for longer than DM− patients (median: 10 days, IQR 8–14 days, vs. median: 9 days, IQR: 6–13 days respectively) (Fig. 2b). DM+ patients have increased rates of ICU admission within 28 days (DM+: 12% (163/1389), DM−: 5% (429/7972)) and death from any cause within 180 days post enrolment as compared to DM− (DM+: 13% (292/2310), DM−: 7% (766/11,301)) (Fig. 2c, d). In a logistic regression with baseline characteristics and comorbidities as predictors, DM had an adjusted Odd’s ratio of 1.56 (1.26–1.93) for ICU admission and 1.31 (1.11–1.54) for death (Supplementary Table 2). Clinical outcome parameters in the DM2 subcohort were similar to the findings in the main cohort (Supplementary Fig. 1a–d).

### Inflammatory response and organ dysfunction

**Fig. 3:**
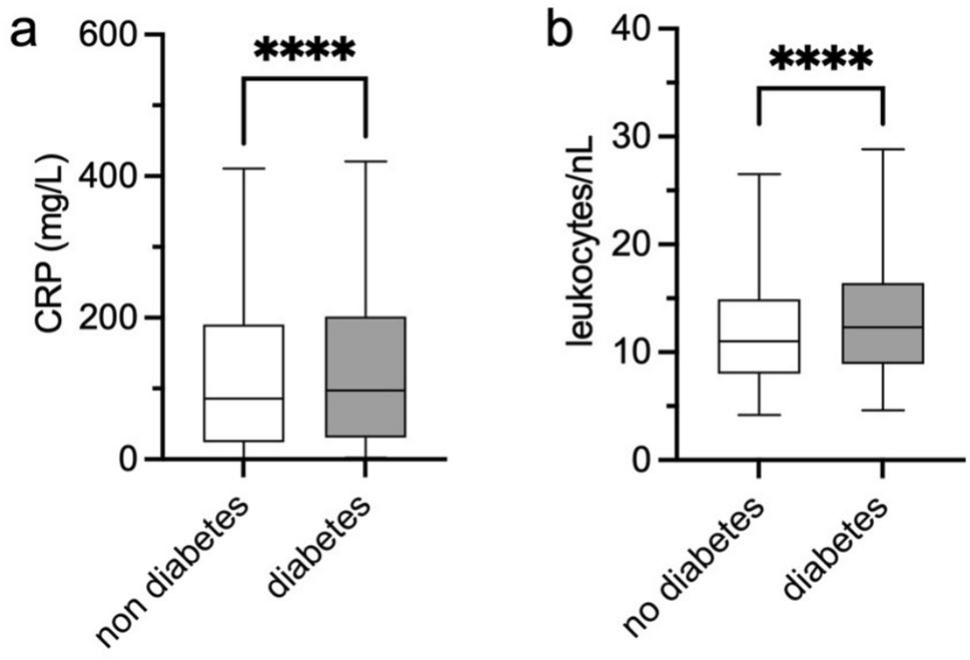
Inflammatory response in DM− and DM+ patients. Box plots representing CRP (**a**) and leucocyte (**b**) values for the baseline blood sample taken within the first 48 h after enrolment in patients without (DM−) and with a history of diabetes mellitus (DM+). Whiskers represent the 2.5–97.5 percentiles. Mann–Whitney test was performed. Data were available for 12,878/13,611 patients for (**a**) and for 12,940/13,611 patients for (**b**). ****: *p* < 0.0001. CRP: C-reactive protein

While CRP and leukocyte values were elevated in both groups at enrolment, they were significantly higher in DM+ compared to DM− patients, although the differences are minimal (Fig. 3a,b). In the ICU DM2 subcohort (see methods), lactate levels and creatinine levels were significantly higher in DM2 + patients compared to DM− patients while other parameters of organ dysfunction did not differ (Supplementary Fig. 2a–f).

### Pathogen spectrum in DM− versus DM+ patients

**Fig. 4:**
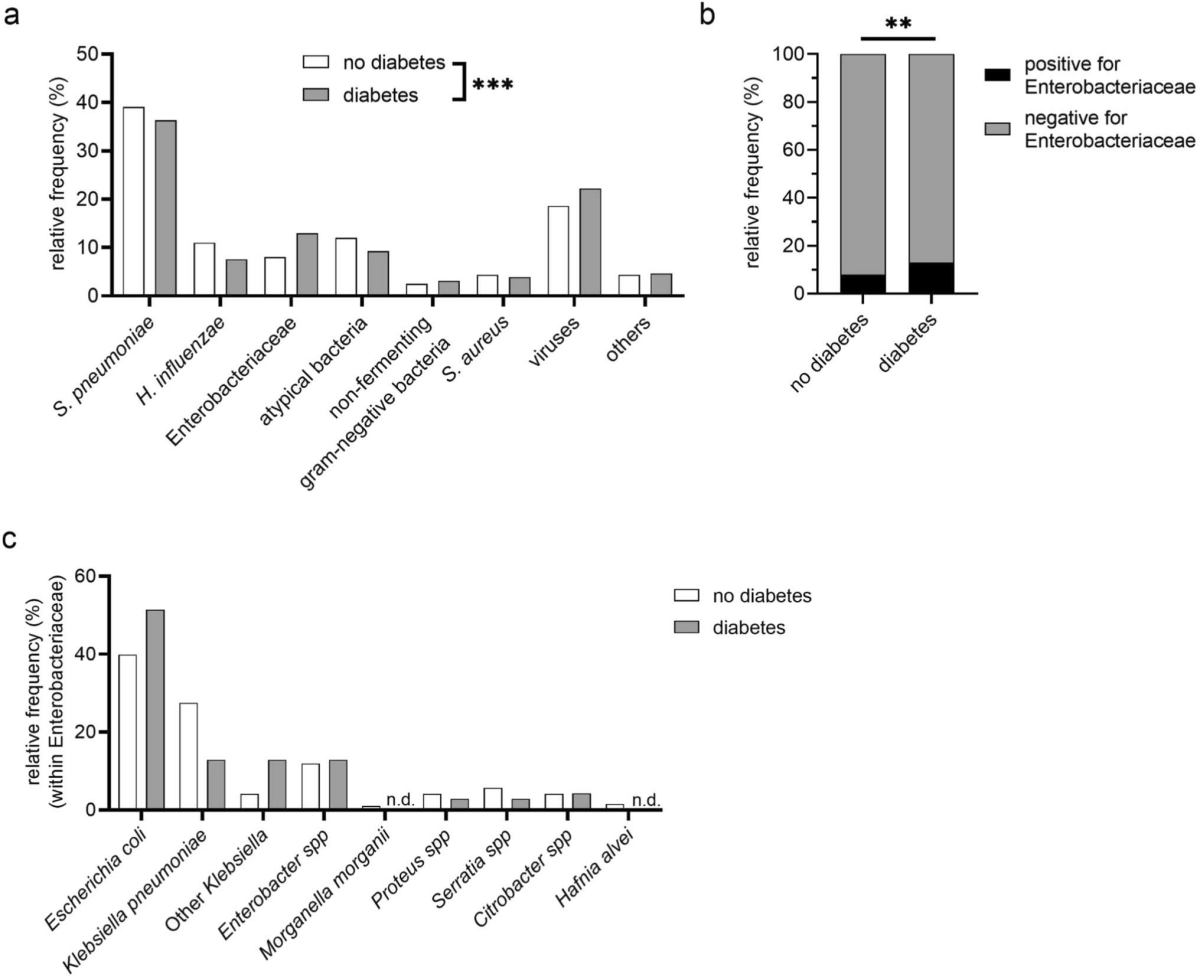
Relative frequencies of identified pathogens in DM− vs DM+ patients. **a** Relative frequencies of identified pathogens in patients without (DM−) and with a history of diabetes mellitus (DM+). Detected pathogens were grouped into seven categories and “other”. Chi-square analysis was performed comparing the overall pathogen distribution between DM− vs DM+ patients. **b** Relative frequency of patients with or without detected Enterobacteriaceae versus all other pathogens grouped in DM− vs DM+ patients. **c** Species distribution within the Enterobacteriaceae group in DM− and DM+ patients. For **a** and **b**, a Chi-square analysis was performed between both study groups and the p value in **b** was adjusted for multiple comparisons (see methods). **: *p* < 0.01; ***: *p* < 0.001; n.d.: not detected

Information on a causative pathogen for CAP was accessible in 21.7% (2954/13,611) of all patients, and in 21.4% (2414/11,301) of DM− and 23.4% (540/2310) of DM+ patients (*p* = 0.03). With respect to the evolution of diagnostic methods over time, we observed a marked increase in pathogen detection rates from the pre-molecular enrolment period before 2017 (2171/11,723; 18.5%) to the era after the establishment of molecular techniques such as PCR (from 2017 onwards; 783/1888; 41.5%). Importantly, there were no significant differences in detection rates between DM+ and DM− patients within either time period (≤2016: DM+ 20.0% (389/1941) vs. DM− 18.2% (1782/9782); ≥2017: DM+ 40.9% (151/369) vs. DM− 41.6% (632/1519)). We observed an overall difference in pathogen distribution in DM− versus DM+ patients (*p* < 0.001, Fig. 4a). *S. pneumoniae* was the most frequently identified pathogen in both groups with 38.5% (1140/2954) overall, 39.1% (944/2414) in DM− patients, and 36.3% (196/540) in DM+ patients. *H. influenzae* accounted for 10.4% (307/2954) overall, 11.0% (266/2414) in DM− patients, and 7.6% (41/540) in DM+ patients. Atypical bacteria were detected in 11.5% (340/2954) of all cases, in 12.0% (290/2414) of DM− patients, and in 9.3% (50/540) of DM+ patients. Enterobacteriaceae accounted for 8.9% (264/2954) of all isolated pathogens in all, 8.0% (194/2414) in DM− and 13.0% (70/540) in DM+ patients. This difference was statistically significant when comparing the relative frequency of Enterobacteriaceae to that of all other pathogens between the groups (*p*_adj_ < 0.005, Fig. 4b). A detailed pathogen distribution from the Enterobacteriaceae group showed that *Escherichia coli* was the most frequently isolated pathogen in both DM− and DM+ patients, but no further analysis was feasible (Fig. 4c). *S. aureus* was isolated in 4.3% (126/2954) of all patients, 4.4% (105/2414) of DM− and 3.9% (21/540) in DM+. The non-fermenting gram-negative bacterial group including *Pseudomonas spp.* and *Acinetobacter baumannii* accounted for 2.6% (78/2954) of all identified pathogens, 2.5% (61/2414) of DM− patients and 3.2% (17/540) of DM+ patients. Viruses were detected in 19.3% (569/2954) of all CAP patients, in 18.6% (449/2414) of DM− and in 22.2% (120/540) of DM+ patients. The “others” pathogen group (see methods) accounted for 4.4% (130/2954) of all isolated pathogens, with 4.4% (105/2414) in DM−, and 4.6% (25/540) in DM+ patients. Except for the difference in Enterobacteriaceae, no significant differences were observed between DM− and DM+ patients when analysing the distribution of every pathogen group against all other pathogens when adjusting for multiple comparisons (Supplementary Table 3). When examining the evolution of the pathogen spectrum across diagnostic eras, we observed some shifts in CAP etiology over time, including a relative decrease in *S. pneumoniae* and an increase in viral detection. Although statistical testing within individual eras was limited by small case numbers, significant differences in the overall pathogen distribution between DM+ and DM− patients were still detectable within each diagnostic era (*p* < 0.05) (Supplementary Fig. 3a,b).

Similar to the observations in the main cohort, we observed a trend towards a lower relative frequency of *S. pneumoniae* and a higher frequency of Enterobacteriaceae in DM+ as compared with DM− patients in the HbA1c subcohort. For the aforementioned pathogen groups, prediabetic patients showed frequencies in between DM− and DM+ patients (Supplementary Fig. 3c).

## Discussion

In this descriptive analysis of a prospective, observational European cohort of patients with CAP, we identified relevant differences between patients with and without DM regarding microbial aetiology, inflammatory parameters, and clinical outcomes.

DM+ patients presented a distinct clinical profile at baseline, including older age and a greater burden of comorbidities, such as cardiovascular disease, chronic kidney disease, and obesity—all of which have independently been associated with worse outcomes in CAP [14]. Patients with DM had higher rates of adverse clinical outcomes, as indicated by higher ICU admission rates and increased 180-day mortality following CAP. DM+ patients were less frequently treated with antibiotics before enrolment into the study, potentially reflecting earlier hospital presentations or a lower likelihood of outpatient treatment. The clinical profile of patients with DM in our cohort is consistent with previous studies [22, 23].

Our first key finding is that the pathogen spectrum in DM+ patients differed significantly from that in DM− patients. Notably, DM+ individuals showed higher relative frequencies of bacteria belonging to the Enterobacteriaceae family. This pathogen group ranked as the second most frequently identified in DM+ patients—after *S. pneumoniae* and ahead of *H. influenzae*. The pathogen detection rate of 21.7% across all patients reflects typical clinical settings [3, 4] and is consistent with earlier CAPNETZ reports [19]. An increased prevalence of Enterobacteriaceae has previously been described for other comorbidities including cardiac, cerebrovascular, respiratory and kidney diseases, but, to the best of our knowledge, our study is the first showing this association with DM [6, 7]. A generally increasing prevalence of this uncommon pathogen group has previously been described for the whole CAPNETZ cohort over time [19]. It also aligns with global estimates showing an increase in pathogens belonging to the Enterobacteriaceae family in patients across all ages with LRIs, along with a decrease in, i.e., *S. pneumoniae *[2], suggesting a broader epidemiological shift in CAP aetiology.

Our second key finding is that DM+ patients exhibited an enhanced inflammatory response at enrolment, as evidenced by higher median CRP levels and leukocyte counts compared to DM− patients. In addition, we observed higher median levels of creatinine and lactate in an ICU subcohort, indicating kidney and cardiovascular impairment in severe CAP patients. However, these findings should be interpreted with caution due to low sample size and lacking information of the baseline in both groups. Earlier mechanistic studies described altered innate immunity in DM, which highlights the need to further investigate immune-pathophysiological pathways in CAP [24, 25].

A strength of our study lies in the well-characterised cohort, which is one of the largest prospective CAP cohorts world-wide [26]. There are, however, limitations to consider: the cohort consisted of mostly hospitalised patients with a predominantly mild to moderate course of CAP. According to the study protocol, DM diagnosis was based on self-report, which may have led to misclassification and a potential underestimation of DM prevalence. Mortality at discharge or at 30 days was not routinely assessed. Pathogen identification was conducted in local laboratories and detection techniques have changed over the 20-year observation period, leading to higher rates of pathogen identification with the introduction of PCR after 2016. During the observation period, the pathogen spectrum itself has changed, and so may have prevalence of diabetes, potentially biasing our findings.

Different studies have reported associations between clinical factors—such as age, functional status, prior hospitalization, antimicrobial therapy, immunosuppressive conditions, and comorbidities—and the occurrence of MDR pathogens and/or Enterobacteriaceae and non-fermenting gram-negative bacilli in CAP [6, 7, 27]. In addition, previous infection with extended-spectrum beta-lactamase–producing organisms, underweight, cardiovascular disease, and prior hospitalization have specifically been linked to Enterobacteriaceae in CAP [28]. Notably, pathogen grouping and the identified risk factors vary considerably across studies. Moreover, diabetes type, antidiabetic medication, and glycemic control may also influence the pathogen spectrum and/or clinical outcomes [29]. However, we were unable to assess these factors due to limited data on diabetes type, lack of detailed information on glycemic control (e.g., serial glucose measurements), and the small number of identified pathogens in some subgroups. Reassuringly, we observed the same trend regarding pathogen distribution in a subcohort with available HBA1c values and differences in clinical outcomes were observed not only in the overall cohort but also in a DM2 subcohort. Of note, a previous analysis of the CAPNETZ study found that patients with high glycemic gap have an increased 90-day mortality [30], raising the interest for further evaluation of different outcomes like CAP causative pathogens according to HbA1c levels. The clinical meaning of the higher inflammatory response observed in our analysis in DM patients, as well as underlying mechanisms, i.e. the potential influence of pneumonia severity on these biomarkers, are unclear at this stage.

In spite of the limitations, we believe that our data, showing a higher frequency of Enterobacteriaceae in DM+ patients, are robust and plausible in light of previous publications with similar observations in comorbidities associated with DM. It might be reasonable to assume that the higher frequency of CAP caused by the Enterobacteriaceae family could be related to compromised early antibacterial immune responses in DM patients [12, 31–35]. The lower prevalence of *S. pneumoniae* in DM patients may be associated with higher vaccination rates in patients with comorbidities following national recommendations [36], thereby relatively increasing the proportion of less common pathogens in the microbiological spectrum.

Further studies are needed to decipher the clinical meaning and impact of the distinct microbial spectrum and of the enhanced inflammatory response we observed in diabetic CAP patients. Given the increasing prevalence of Enterobacteriaceae, clinical risk factors for CAP with this pathogen group should be reexamined in larger studies. To date, current CAP guidelines such as the French [37] and German [38], state that pathogen testing can be omitted in patients with CAP not necessitating hospital admission, i.e. with mild pneumonia. While complexity of testing and procedures, cost-effectiveness and low levels of pathogen detection remain a concern in the outpatient setting, based on our and other group’s findings, we propose extending current recommendations to include early, comprehensive, and ideally rapid microbiological testing in at-risk patients with chronic underlying conditions— even in the absence of traditional indicators for hospital admission or microbial diagnostics. When weighing the pros and cons of microbial testing in mild CAP, it is important to consider that a shift in CAP aetiology has been observed in our and other studies, particularly in patients with interrelated comorbidities such as DM. In these patients, Enterobacteriaceae and viral pathogens are increasingly replacing traditionally expected CAP pathogens. Some of the genera belonging to the Enterobacteriaceae have limited susceptibility to aminopenicillines and first-line empirical treatment may therefore not be effective in these patients. Early pathogen identification is essential for enabling timely adjustment or discontinuation of antibiotic therapy, which has been shown to improve CAP outcomes and is critical for avoiding both under- and overtreatment [5, 39]. While at this stage no conclusion for clinical management other than early and complete pathogen testing including molecular tests leading to timely available results can be drawn, our studies point to mechanisms that merit further investigation. For example, the impact of diagnostic and antibiotic stewardship, as well as the adequacy of antibiotic therapy, could be examined in large CAP populations, particularly among patients at risk for uncommon pathogens. RCTs could investigate the potential benefit of another first-line empirical therapy in CAP patients with risk factors for Enterobacteriaceae in which a viral cause has been ruled out, as well as the potential benefit of antiinflammatory medications including steroids in this specific patient population. Recent guidelines recommend the use of steroids in the setting of severe CAP in order to counterbalance a dysregulated inflammatory response [40]. To our knowledge, data on the impact of steroids in this particular patient group are scarce and would be of great interest, as corticosteroids are well-known to induce hyperglycemia as a side-effect, which may in turn contribute to adverse outcomes.

Taken together, our findings underscore that patients with DM represent a highly prevalent, specific, and clinically vulnerable population in the context of CAP—highlighting the need for increased awareness among healthcare professionals, appropriate consideration in CAP guidelines, and further research aiming at optimising treatment recommendations and reducing adverse outcomes.

## Supplementary Information

Below is the link to the electronic supplementary material.

Supplementary file1 (DOCX 580 KB)

## Data Availability

No datasets were generated or analysed during the current study.

## References

[1] https://www.who.int/news-room/fact-sheets/detail/the-top-10-causes-of-death. Accessed 12 June 2025.

[2] GBD 2021 Lower Respiratory Infections and Antimicrobial Resistance Collaborators. Global, regional, and national incidence and mortality burden of non-COVID-19 lower respiratory infections and aetiologies, 1990–2021: a systematic analysis from the Global Burden of Disease Study 2021. Lancet Infect Dis 2024;24:974–1002. 10.1016/S1473-3099(24)00176-2 PMC11339187 38636536

[3] Gadsby NJ, Musher DM. The microbial etiology of community-acquired pneumonia in adults: from classical bacteriology to host transcriptional signatures. Clin Microbiol Rev. 2022;35:e0001522. 36165783 10.1128/cmr.00015-22 PMC9769922

[4] Jain S, Self WH, Wunderink RG, Fakhran S, Balk R, Bramley AM, et al. Community-acquired pneumonia requiring hospitalization among U.S. adults. N Engl J Med. 2015;373:415–27. 26172429 10.1056/NEJMoa1500245 PMC4728150

[5] Vaughn VM, Dickson RP, Horowitz JK, Flanders SA. Community-acquired pneumonia: a review. JAMA. 2024;332:1282–95. 39283629 10.1001/jama.2024.14796

[6] von Baum H, Welte T, Marre R, Suttorp N, Ewig S, CAPNETZ study group. Community-acquired pneumonia through Enterobacteriaceae and Pseudomonas aeruginosa: Diagnosis, incidence and predictors. Eur Respir J 2010;35:598–605. 10.1183/09031936.00091809 19679601

[7] Aliberti S, Di Pasquale M, Zanaboni AM, Cosentini R, Brambilla AM, Seghezzi S, et al. Stratifying risk factors for multidrug-resistant pathogens in hospitalized patients coming from the community with pneumonia. Clin Infect Dis. 2012;54(4):470–8. 22109954 10.1093/cid/cir840

[8] Benfield T, Jensen JS, Nordestgaard BG. Influence of diabetes and hyperglycaemia on infectious disease hospitalisation and outcome. Diabetologia. 2007;50:549–54. 17187246 10.1007/s00125-006-0570-3

[9] Almirall J, Bolíbar I, Serra-Prat M, Roig J, Hospital I, Carandell E, et al. New evidence of risk factors for community-acquired pneumonia: a population-based study. Eur Respir J. 2008;31:1274–84. 18216057 10.1183/09031936.00095807

[10] Jensen AV, Faurholt-Jepsen D, Egelund GB, Andersen SB, Petersen PT, Benfield T, et al. Undiagnosed diabetes mellitus in community-acquired pneumonia: a prospective cohort study. Clin Infect Dis. 2017;65:2091–8. 29095981 10.1093/cid/cix703

[11] Muller LMAJ, Gorter KJ, Hak E, Goudzwaard WL, Schellevis FG, Hoepelman AIM, et al. Increased risk of common infections in patients with type 1 and type 2 diabetes mellitus. Clin Infect Dis. 2005;41:281–8. 16007521 10.1086/431587

[12] Chávez-Reyes J, Escárcega-González CE, Chavira-Suárez E, León-Buitimea A, Vázquez-León P, Morones-Ramírez JR, et al. Susceptibility for some infectious diseases in patients with diabetes: the key role of glycemia. Front Public Health. 2021;9:559595. 33665182 10.3389/fpubh.2021.559595 PMC7921169

[13] Barmanray RD, Cheuk N, Fourlanos S, Greenberg PB, Colman PG, Worth LJ. In-hospital hyperglycemia but not diabetes mellitus alone is associated with increased in-hospital mortality in community-acquired pneumonia (CAP): a systematic review and meta-analysis of observational studies prior to COVID-19. BMJ Open Diabetes Res Care. 2022;10:e002880. 35790320 10.1136/bmjdrc-2022-002880 PMC9257863

[14] Falguera M, Pifarre R, Martin A, Sheikh A, Moreno A. Etiology and outcome of community-acquired pneumonia in patients with diabetes mellitus. Chest. 2005;128:3233–9. 16304267 10.1378/chest.128.5.3233

[15] Klekotka RB, Mizgała E, Król W. The etiology of lower respiratory tract infections in people with diabetes. Pneumonol Alergol Pol. 2015;83:401–8. 26379004 10.5603/PiAP.2015.0065

[16] Capnetz – Ziel und Zweck der CAPNETZ STIFTUNG ist die Förderung wissenschaftlicher Aktivitäten rund um das Thema „Ambulant erworbene Pneumonien (community-acquired pneumonia, CAP) und andere Infektionen des unteren Respirationstraktes“ n.d. http://www.capnetz.de. Accessed 19 June 2025.

[17] Welte T, Suttorp N, Marre R. CAPNETZ-community-acquired pneumonia competence network. Infection. 2004;32(4):234–8. 15293080 10.1007/s15010-004-3107-z

[18] Bauer TT, Ewig S, Marre R, Suttorp N, Welte T, CAPNETZ Study Group. CRB-65 predicts death from community-acquired pneumonia. J Intern Med. 2006;260:93–101. 16789984 10.1111/j.1365-2796.2006.01657.x

[19] Braeken DCW, Essig A, Panning M, Hoerster R, Nawrocki M, Dalhoff K, et al. Shift in bacterial etiology from the CAPNETZ cohort in patients with community-acquired pneumonia: data over more than a decade. Infection. 2021;49:533–7. 33774804 10.1007/s15010-021-01605-w PMC8159805

[20] Krüger S, Ewig S, Papassotiriou J, Kunde J, Marre R, von Baum H, et al. Inflammatory parameters predict etiologic patterns but do not allow for individual prediction of etiology in patients with CAP: results from the German competence network CAPNETZ. Respir Res. 2009;10:65. 19594893 10.1186/1465-9921-10-65 PMC2714042

[21] American Diabetes Association Professional Practice Committee. 2. Diagnosis and classification of diabetes: standards of care in diabetes-2025. Diabetes Care 2025;48:S27–49. 10.2337/dc25-S002 PMC11635041 39651986

[22] Huang C, Wang Y, Li X, Ren L, Zhao J, Hu Y, et al. Clinical features of patients infected with 2019 novel coronavirus in Wuhan, China. Lancet. 2020;395:497–506. 31986264 10.1016/S0140-6736(20)30183-5 PMC7159299

[23] López-de-Andrés A, de Miguel-Díez J, Jiménez-Trujillo I, Hernández-Barrera V, de Miguel-Yanes JM, Méndez-Bailón M, et al. Hospitalisation with community-acquired pneumonia among patients with type 2 diabetes: an observational population-based study in Spain from 2004 to 2013. BMJ Open. 2017;7:e013097. 28057653 10.1136/bmjopen-2016-013097 PMC5223662

[24] Lachmandas E, Vrieling F, Wilson LG, Joosten SA, Netea MG, Ottenhoff TH, et al. The effect of hyperglycaemia on in vitro cytokine production and macrophage infection with *Mycobacterium tuberculosis*. PLoS ONE. 2015;10:e0117941. 25664765 10.1371/journal.pone.0117941 PMC4322041

[25] Dungu AM, Ryrsø CK, Hegelund MH, Jensen AV, Kristensen PL, Krogh-Madsen R, et al. Diabetes status, c-reactive protein, and insulin resistance in community-acquired pneumonia-a prospective cohort study. J Clin Med. 2023. 10.3390/jcm13010245. 38202252 10.3390/jcm13010245 PMC10780000

[26] Suttorp N, Welte T, Marre R, Stenger S, Pletz M, Rupp J, et al. CAPNETZ. The competence network for community-acquired pneumonia (CAP): Das Kompetenzzentrum für ambulant erworbene Pneumonie. Bundesgesundheitsblatt Gesundheitsforschung Gesundheitsschutz 2016;59:475–81. 10.1007/s00103-016-2318-7 26984399

[27] Nakagawa N, Katsurada M, Fukuda Y, Noguchi S, Horita N, Miki M, et al. Risk factors for drug-resistant pathogens in community-acquired pneumonia: systematic review and meta-analysis. Eur Respir Rev. 2025;34:240183. 40107661 10.1183/16000617.0183-2024 PMC11920891

[28] Villafuerte D, Aliberti S, Soni NJ, Faverio P, Marcos PJ, Wunderink RG, et al. Prevalence and risk factors for Enterobacteriaceae in patients hospitalized with community-acquired pneumonia. Respirology. 2020;25:543–51. 31385399 10.1111/resp.13663

[29] Kantreva K, Katsaounou P, Saltiki K, Trakada G, Ntali G, Stratigou T, et al. The possible effect of anti-diabetic agents GLP-1RA and SGLT-2i on the respiratory system function. Endocrine. 2025;87:378–88. 39289244 10.1007/s12020-024-04033-6

[30] Jensen AV, Baunbæk Egelund G, Bang Andersen S, Petersen PT, Benfield T, Witzenrath M, et al. The glycemic gap and 90-day mortality in community-acquired pneumonia. A prospective cohort study. Ann Am Thorac Soc. 2019;16:1518–26. 31437014 10.1513/AnnalsATS.201901-007OC

[31] Restrepo BI, Twahirwa M, Rahbar MH, Schlesinger LS. Phagocytosis via complement or Fc-gamma receptors is compromised in monocytes from type 2 diabetes patients with chronic hyperglycemia. PLoS ONE. 2014;9:e92977. 24671137 10.1371/journal.pone.0092977 PMC3966862

[32] Fiocca Vernengo F, Röwekamp I, Boillot L, Caesar S, Dörner PJ, Tarnowski B, et al. Diabetes impairs IFNγ-dependent antibacterial defense in the lungs. Mucosal Immunol. 2025;18:431–40. 39746547 10.1016/j.mucimm.2024.12.015

[33] Lachmandas E, Thiem K, van den Heuvel C, Hijmans A, de Galan BE, Tack CJ, et al. Patients with type 1 diabetes mellitus have impaired IL-1β production in response to *Mycobacterium tuberculosis*. Eur J Clin Microbiol Infect Dis. 2018;37:371–80. 29189980 10.1007/s10096-017-3145-y PMC5780542

[34] Tripathi D, Radhakrishnan RK, Sivangala Thandi R, Paidipally P, Devalraju KP, Neela VSK, et al. IL-22 produced by type 3 innate lymphoid cells (ILC3s) reduces the mortality of type 2 diabetes mellitus (T2DM) mice infected with Mycobacterium tuberculosis. PLoS Pathog. 2019;15:e1008140. 31809521 10.1371/journal.ppat.1008140 PMC6919622

[35] Lecube A, Pachón G, Petriz J, Hernández C, Simó R. Phagocytic activity is impaired in type 2 diabetes mellitus and increases after metabolic improvement. PLoS ONE. 2011;6:e23366. 21876749 10.1371/journal.pone.0023366 PMC3158070

[36] https://www.rki.de/DE/Aktuelles/Publikationen/Epidemiologisches-Bulletin/2025/04_25.pdf?__blob=publicationFile&v=10. Accessed 19 June 2025.

[37] Dinh A, Barbier F, Bedos J-P, Blot M, Cattoir V, Claessens Y-E, et al. Update of guidelines for management of community acquired pneumonia in adults by the French Infectious Disease Society (SPILF) and the French-Speaking Society of Respiratory Diseases (SPLF): endorsed by the French intensive care society (SRLF), the French microbiology society (SFM), the French radiology society (SFR) and the French emergency society (SFMU). Respir Med Res. 2025;87:101161. 40037948 10.1016/j.resmer.2025.101161

[38] Ewig S, Kolditz M, Pletz M, Altiner A, Albrich W, Drömann D, et al. Behandlung von erwachsenen Patienten mit ambulant erworbener Pneumonie – Update 2021. Pneumologie. 2021;75:665–729. 34198346 10.1055/a-1497-0693

[39] Uematsu H, Hashimoto H, Iwamoto T, Horiguchi H, Yasunaga H. Impact of guideline-concordant microbiological testing on outcomes of pneumonia. Int J Qual Health Care. 2014;26:100–7. 24257160 10.1093/intqhc/mzt078

[40] Chaudhuri D, Nei AM, Rochwerg B, Balk RA, Asehnoune K, Cadena R, et al. 2024 focused update: Guidelines on use of corticosteroids in sepsis, acute respiratory distress syndrome, and community-acquired pneumonia. Crit Care Med. 2024;52:e219–33. 38240492 10.1097/CCM.0000000000006172

