# Supplementary material for: Community-acquired pneumonia in diabetic patients is characterised by a distinct pathogen spectrum and enhanced inflammation: results from CAPNETZ, a prospective observational cohort study
Source: Infection. 2025 Oct 12;54(1):275–85. doi: 10.1007/s15010-025-02659-w (PMC12864321; doi:10.1007/s15010-025-02659-w)
Supplement: Supplementary file 1 — Supplementary file1 (DOCX 580 KB) [file 15010_2025_2659_MOESM1_ESM.docx]

## **Supplementary Table 1 Clinical characteristics of a subcohort of CAPNETZ admitted between 2017-2022 including all, type 2 diabetic and non-diabetic patients.**

|  | **All patients** | **Type 2 Diabetes mellitus** | **Non-diabetic** | **p-value** |
| --- | --- | --- | --- | --- |
| % (n/N) | 100 (1692) | 19 (317/1692) | 81 (1375/1692) | - |
| Age in years (Mean (SD)) | 64 (17) | 72 (12) | 62 (17) | <0,0001 |
| Male Sex, (% (n/ N)) | 63 (1085/1692) | 66 (208/317) | 63 (860/1375) | 0,30 |
| BMI (kg/m2), Median (IQR), available n | 26 (23-30) | 29 (25-34) | 26 (23-29) | <0,0001 |
| Nursing home residency (%, n/available N) | 4 (61/1690) | 9 (28/317) | 2 (33/1373) | <0,0001 |
| Cigarette smoking during the last 12 months (%, n/available N) | 23 (368/1625) | 16 (47/303) | 24 (321/1322) | <0,01 (0,001) |
| Chronic heart failure (%, n/available N) | 7 (112/1692) | 14 (44/317) | 5 (68/1375) | <0,0001 |
| Chronic respiratory/pulmonary disease (%, n/available N) | 35 (595/1692) | 41 (130/317) | 34 (465/1375) | <0,05 (0,0156) |
| Vascular/Cerebrovascular disease (%, n/available N) | 59 (996/1692) | 89 (282/317) | 52 (714/1375) | <0,0001 |
| Chronic kidney disease (%, n/available N) | 13 (217/1692) | 26 (83/317) | 10 (134/1375) | <0,0001 |
| Previous antibiotic therapy within the last 4 weeks (%, n/available N) | 13 (223/1692) | 8 (24/317) | 14 (199/1375) | <0,01 (0,001) |
| Malignant disease | 16 (274/1692) | 17 (53/317) | 16 (221/1375) | 0,78 |
| Oxygen Therapy | 3 (51/1692) | 4 (14/317) | 3 (37/1375) | 0,10 |

Available n is indicated where variables were not available for all patients. P-values comparing type 2 diabetic and non-diabetic patients (based on self-reporting) were calculated using the Wilcoxon test for medians after rolling out a normal distribution, and unadjusted Chi-square test for frequencies of comorbidities. All clinical variables shown were significantly different between diabetic and non-diabetic patients except for gender, malignant disease and oxygen therapy . **Abbreviation**s : IQR : interquartile range, SD: standard deviation, BMI : body mass index.

# **Supplementary Table 2 Multivariate logistic regression model of outcome including demographic characteristics and comorbidities as predictors**

|  | **Outcome ICU admission** | | **Outcome death** | |
| --- | --- | --- | --- | --- |
| **Predictors** | **aOR** | **95% CI** | **aOR** | **95% CI** |
| **Age** | 1.01 | 1.00, 1.02 | 1.05 | 1.05, 1.06 |
| **Male sex at birth** | 1.73 | 1.44, 2.10 | 1.56 | 1.35, 1.82 |
| **BMI** | 1.01 | 1.00, 1.03 | 0.93 | 0.91, 0.94 |
| **Heart disease** | 1.51 | 1.20, 1.89 | 1.78 | 1.52, 2.08 |
| **Cerebrovascular disease** | 1.50 | 1.21, 1.85 | 1.50 | 1.28, 1.76 |
| **Kidney disease** | 1.59 | 1.24, 2.04 | 1.50 | 1.25, 1.80 |
| **Respiratory disease** | 1.05 | 0.88, 1.25 | 0.98 | 0.85, 1.13 |
| **Malignant disease** | 0.99 | 0.75, 1.29 | 1.91 | 1.60, 2.28 |
| **Diabetes mellitus** | 1.56 | 1.26, 1.93 | 1.31 | 1.11, 1.54 |

Available n/N was 9,168/13,611 for the outcome ICU admission and 13,164/13,611 for the outcome death. Logistic regression on the outcome ICU admission within 28 days versus not and death from any cause within 180 days post enrolment was performed including baseline characteristics and comorbidities as predictors.

Abbreviations: ICU: intensive care unit. aOR: adjusted Odd’s ratio. CI: confidence interval. BMI: body mass index.

##

##

## **Supplementary Table 3 Specific pathogen group comparison between DM- and DM+ patients**

| **Pathogen** | ***P* value** | **Adjusted *P* Value (p_adj_)** |
| --- | --- | --- |
| *S. pneumoniae* | 0.2406 | 0.8894 |
| *H. influenzae* | 0.0191 | 0.1430 |
| Enterobacteriaceae | 0.0006 | 0.0048 |
| Atypical bacteria | 0.0734 | 0.4566 |
| Non-fermenting gram-negative bacteria | 0.4569 | 0.9924 |
| *S. aureus* | 0.7240 | 1.0000 |
| Viruses | 0.0612 | 0.3966 |
| Others | 0.7292 | 1.0000 |

Chi-square test was used to evaluate the individual difference between each pathogen group versus all other pathogens, and the obtained *P* values were adjusted for multiple comparisons with the Bonferroni-Sidak test.


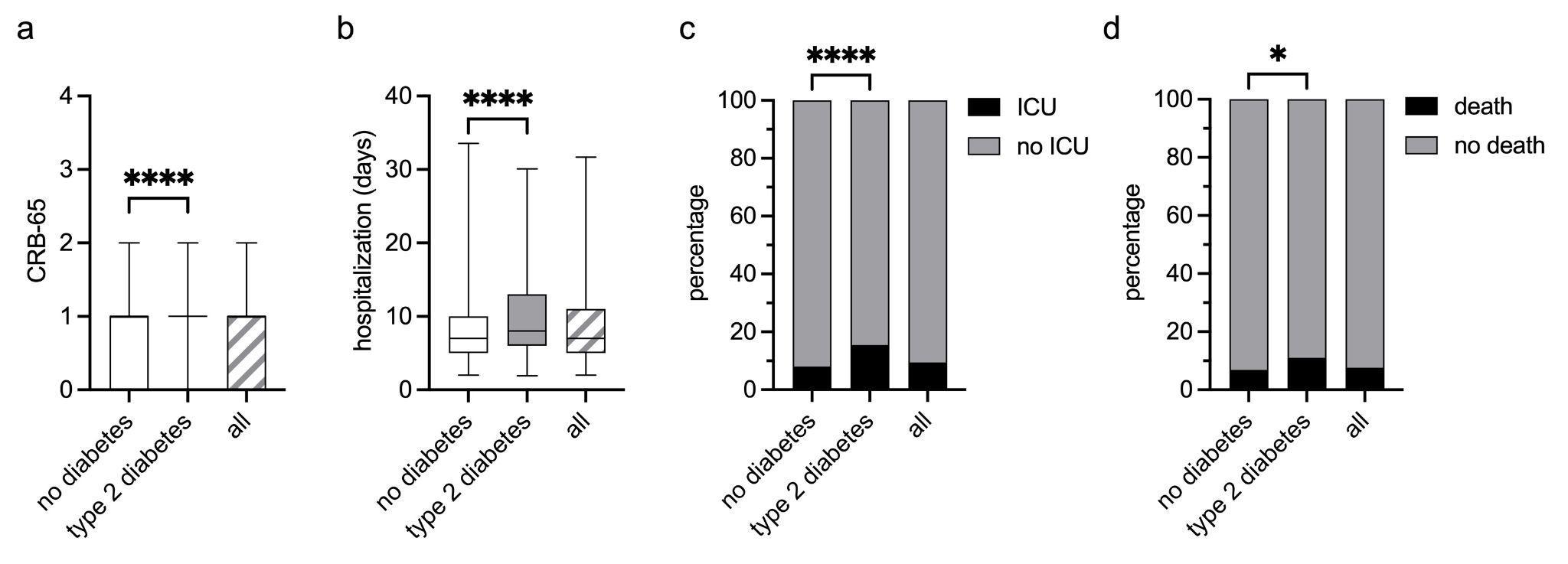
**Supplementary Fig.1 Outcome in non-diabetic versus type 2 diabetic patients from CAPNETZ study Nr. 2**

(a, b) Box-plot showing the distribution (median and IQR) of CRB-65 score (a) and hospitalization days (b) in non-diabetic, type 2 diabetic, and all patients (available 1,516/1,692 for a) and 1,652/1,692 for b)). Whiskers represent the 2,5 - 97,5 percentile. Mann-Whitney statistical analysis was used. (c, d) Percentages of ICU admission within 28 days (c) and death within 180 days (d), both from any cause following CAP in non-diabetic vs type 2 diabetic patients, as well as all patients in the CAPNETZ 2 cohort (available n = 1,688/1,692 for c) and 1,692/1,692 for d)). Chi-square test was used to analyze the shown groups. *: p<0,05 ; ****: p<0,0001. ICU: intensive care unit, CRB-65: pneumonia severity index (see methods). Diabetes Type 2 was defined on a self-report basis.


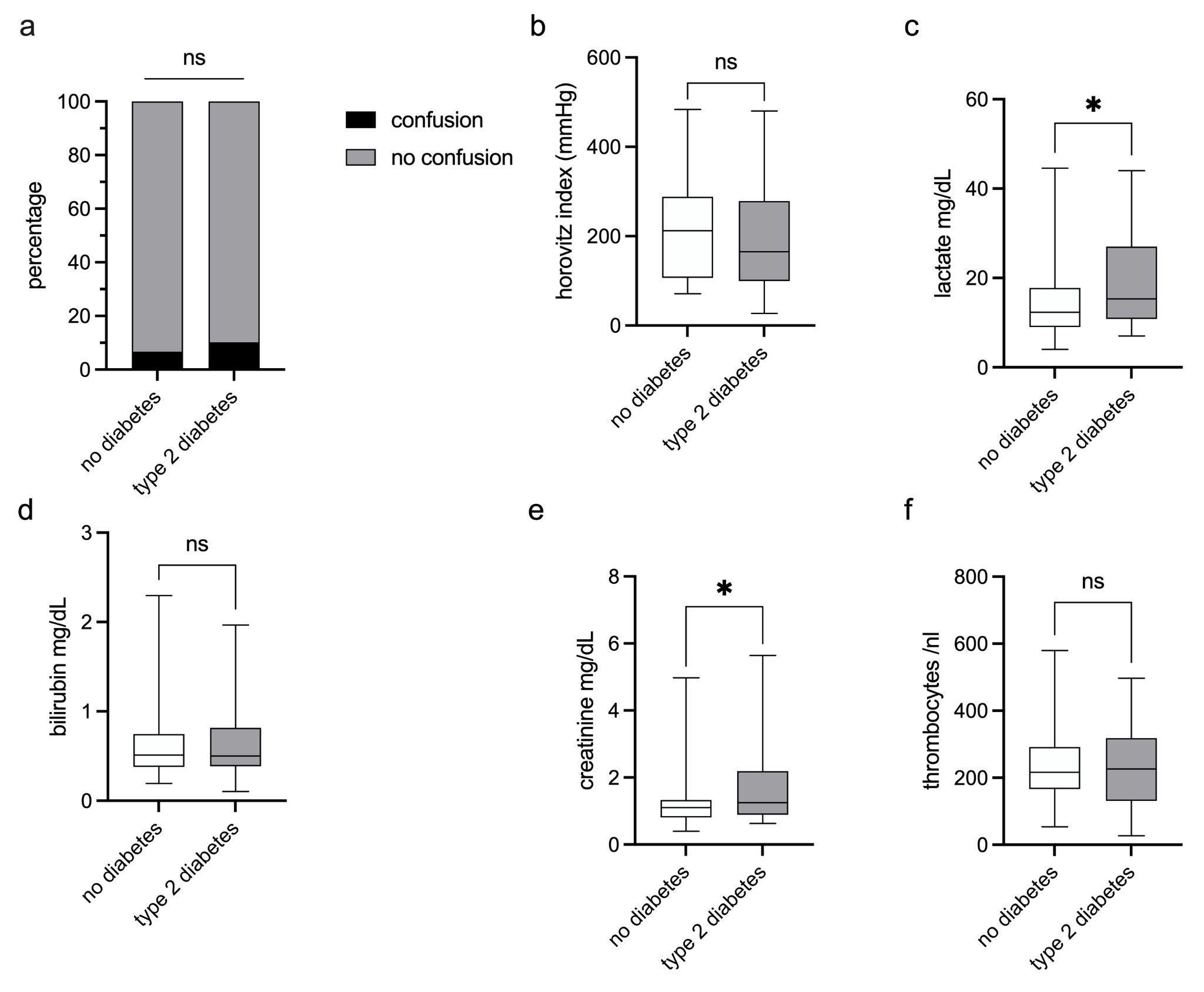


**Supplementary Fig. 2** **Markers for organ failure in non-diabetic versus type 2 diabetic patients admitted in the ICU**

(a) Percentage of confusion at the time of ICU admission in non-diabetic vs. type 2 diabetic patients. Fisher’s exact test was used to analyze the difference between both groups. (b–f) Box plots showing values of the Horovitz index (b), lactate (c), bilirubin (d), creatinine (e), and thrombocytes (f) from baseline blood samples collected within 48 hours after inclusion, comparing non-diabetic and type 2 diabetic patients. Whiskers represent the 2.5th to 97.5th percentiles. After ruling out normal distribution, the Mann–Whitney test was applied. Available data for non-diabetic and type 2 diabetic patients: (a) 105 and 49; (b) 84 and 41; (c) 56 and 31; (d) 93 and 40; (e) 107 and 49. *p < 0.05. The Horovitz index (PaO₂/FiO₂ ratio) reflects pulmonary gas exchange efficiency and is used here as a marker of lung failure severity. Diabetes status and type was determined by self-report. **Abbreviations**: ns = not significant


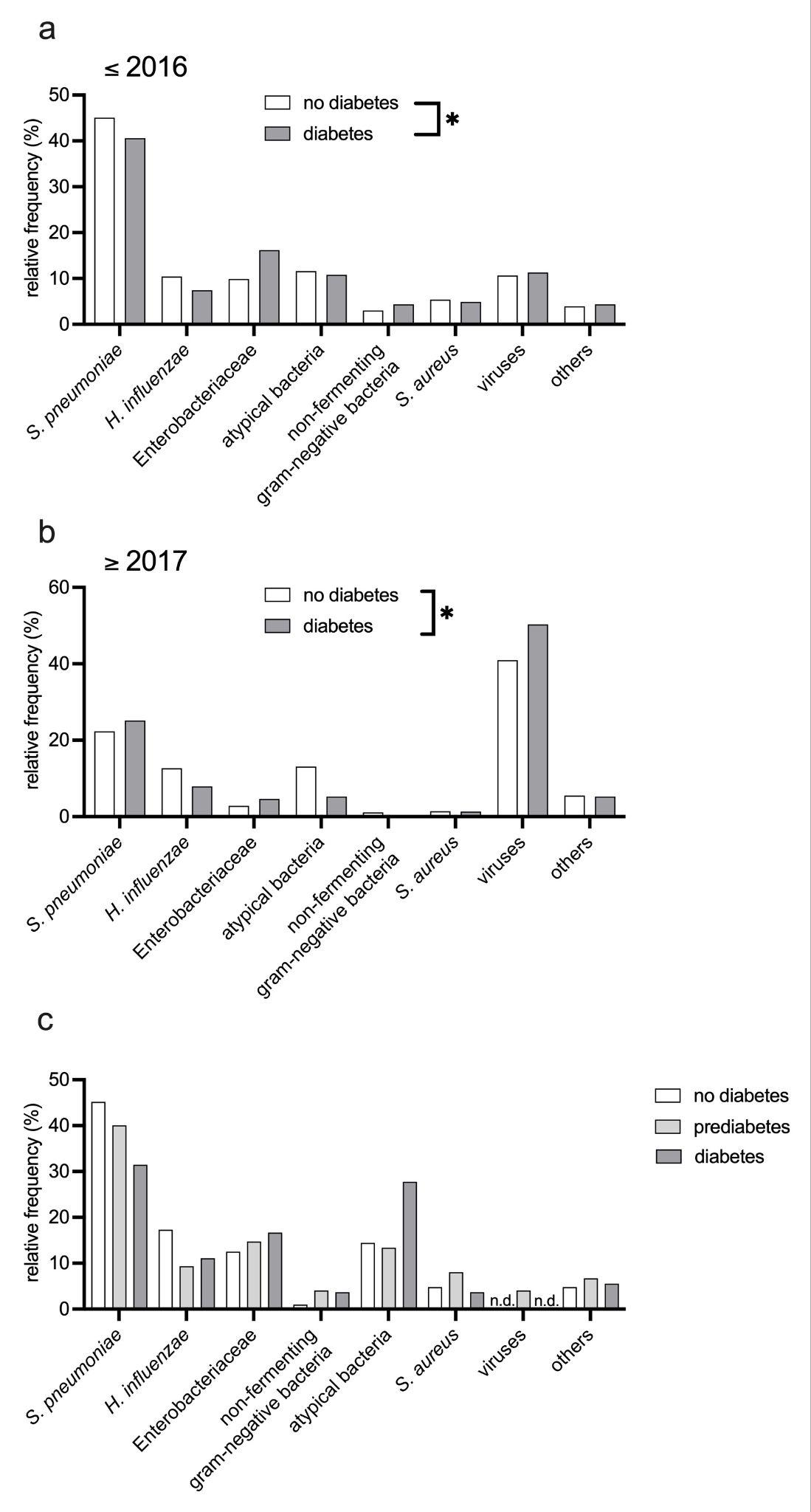


**Supplementary Fig.3 Relative frequencies of isolated pathogens over time and based on self-reporting and HbA1c levels**

(a,b) Relative frequencies of identified pathogens in patients without (DM-) and with a history of diabetes mellitus (DM+) enrolled a) until 2016 (pre-molecular era) and b) from 2017 onwards (establishment of molecular detection methods). Detected pathogens were grouped into seven categories and “other”. Chi-square analysis was performed comparing the overall pathogen distribution between DM- vs DM+ patients. In the era >=2017 the group of “non-fermeting gram-negative bacteria” was excluded from the statistical testing (n=0 in DM+). (c) Pathogen relative frequency summarized in seven biologically relevant groups and “others” in 104 non-diabetic, 75 pre-diabetic, and 54 diabetic patients based on available HbA1c data (see methods). * p<0,05, n.d.: not detected.
